# Remarkable Improvement in the Mechanical Properties of Epoxy Composites Achieved by a Small Amount of Modified Helical Carbon Nanotubes

**DOI:** 10.3390/polym10101103

**Published:** 2018-10-05

**Authors:** Nabil Kadhim, Yuan Mei, Ying Wang, Ying Li, Fanbin Meng, Man Jiang, Zuowan Zhou

**Affiliations:** 1 Key Laboratory of Advanced Technologies of Materials (Ministry of Education), School of Materials Science and Engineering, Southwest Jiaotong University, Chengdu 610031, China; nabiltaieh@gmail.com (N.K.); ywang102@163.com (Y.W.); y.maryli@my.swjtu.edu.cn (Y.L.); mengfanbin_wing@126.com (F.M.); jiangman1021@swjtu.edu.cn (M.J.); 2 Technical College-Baghdad, Middle Technical University, Baghdad 10074, Iraq

**Keywords:** helical carbon nanotubes, epoxy composites, mechanical properties, surface treatment

## Abstract

Helical carbon nanotubes (HCNTs) were functionalized to fabricate HCNT/epoxy composites. Acid oxidation and a silane coupling agent, glycidoxypropyltrimethoxysilane (KH560), were used to modify the HCNTs. Remarkably, the flexural strength and the flexural strain were enhanced by 72.0% and 325.0%, respectively, compared to pure epoxy after adding a small amount of the KH560 modified HCNTs (K-HCNTs). Simultaneously, the tensile strength and Young’s modulus of K-HCNTs/epoxy composites were 51.3% and 270.9% higher than those of pure epoxy. It is found that the presence of silane molecules improved the dispersion of HCNTs in epoxy and the interfacial interaction. Moreover, it has been found that the mechanically interlocking effect from the helical shape of HCNTs also contributes to the improved mechanical properties of epoxy composites, compared to their straight multi-walled carbon nanotube (MWCNT) counterparts. This work provides a low-cost and efficient approach to strengthen and toughen epoxy composites.

## 1. Introduction

Epoxy resins have attracted great attention for wide applications in the aerospace and automobile industry, owing to their excellent mechanical properties, strong bonding strength and good thermal stabilities [1,2]. However, the inherent brittleness of cured epoxy severely limited further applications [3]. In order to overcome these limits, various types of nanofillers, such as silica and rubber particles [4,5,6], clay [7,8] as well as graphene nanoplatelets [9], are incorporated into epoxy. Among them, carbon nanotube (CNT) is a highly promising candidate for reinforcing and toughening epoxy because of its low density, high strength and modulus [10,11,12,13,14]. In addition, significant improvement in the thermal and electrical properties of epoxy composites can also be achieved by adding CNTs [15,16].

To achieve a remarkable enhancement in the mechanical properties of CNT/epoxy composites, two main issues should be deliberately considered. One is that CNTs tend to aggregate in a polymer matrix due to the strong π–π interactions and van de Waal’s forces between them, resulting in the uneven dispersion of CNTs. In addition, the dispersion of filler become worse with increasing filler content owing to the presence of filler agglomerate [17]. Furthermore, the existence of CNT aggregate limits the theoretical enhancement in the mechanical properties of epoxy composites due to a reduction of their effective aspect ratio [18]. The other one is that the poor interfacial interaction between the CNTs and polymer matrix leads to interfacial debonding when the mechanical loading is subjected, which hinders the mechanical stress transfer from the polymer matrix to the CNTs [16]. Therefore, various surface modifications have been attempted to solve the issues above. Kim et al. [19] modified the CNTs by amine or plasma oxidation and found that the surface-treated CNTs are well dispersed in epoxy and have strong interfacial bonding with the matrix, improving the tensile properties of epoxy composites. Yang et al. [1] also reported that the enhanced impact and bending strength are the results of the homogenous dispersion of triethylenetetramine-grafting CNTs in the epoxy and the strong interfacial interactions. In addition to the chemical grafting modification, the nonionic surfactants are also very effective to modify the CNTs for composites without deteriorating sp^2^ bonding of CNTs. It has been found that the flexural strength and the flexural modulus of epoxy composites are increased by the addition of Triton-treated CNTs, owing to the fact that the surfactant serves as an interfacial coupling agent and a dispersant [20].

However, the mechanical properties of CNTs/epoxy composites are still far from expectations despite the extensive studies on surface modification of straight CNTs. The root may be the fact that it is difficult to form the mechanical interlocking at the straight CNT/epoxy interface. However, the curved surface morphology of helical carbon nanotubes (HCNTs) offers an advantage in forming the mechanical interlocking at the interface, which further enhances the interfacial interaction between HCNTs and epoxy. Lau et al. [21] compared the flexural properties of straight CNT and HCNT/epoxy composites. It is found that the contributions of HCNTs to the hardness and the flexural strength of epoxy composites are superior to those of single-wall carbon nanotubes (SWCNTs), owing to the good mechanical interlocking between the coils of HCNTs and epoxy. Subsequently, the nanomechanical properties of HCNT/epoxy composites were systematically explored by Li et al. [22]. The hardness, elastic modulus and tensile strength of HCNT/epoxy composites were significantly enhanced due to the good dispersion of HCNTs and the interlocking between the HCNTs and epoxy. These works suggested the incorporation of pristine HCNTs into epoxy can endow the epoxy composites with better mechanical properties than compared with their straight counterparts. The mechanical properties of composites are expected to be improved further if surface modification of the HCNTs is applied. Unfortunately, to our knowledge, the role of surface-treated HCNTs on the mechanical properties of epoxy composites is rarely reported.

In this work, the effect of surface-treated HCNTs on the flexural and tensile properties of HCNT/epoxy composites is systematically investigated. Two different surface modification methods, namely acid oxidation and silanization, were employed to promote the dispersion of HCNTs in epoxy and enhance the interfacial interaction between the HCNTs and epoxy. In addition, the contributions of straight CNTs and HCNTs on the mechanical properties of epoxy composites were compared to further reveal the strengthening and toughening mechanisms of HCNTs in epoxy.

## 2. Experimental

### 2.1. Materials

A standard bisphenol A epoxy resin (E44) and a common aromatic diamine hardener (curing agent) 4,4′-diaminodiphenylsulfone (DDS) were supplied by Nantong synthetic Materials Co., Ltd., Nantong, China. HCNTs with an average diameter of 80–120 nm and length of 3–5 μm were prepared by chemical vapour deposition (CVD) in the laboratory [23]. The multi-walled carbon nanotubes (MWCNTs) were purchased from Chengdu Organic Chemicals Co. Ltd., Chengdu, China. The diameter of MWCNTs was 50–80 nm, and the length was around 10–20 μm. Tetrahydrofuran (THF), concentrated nitric acid (HNO_3_, 63%), concentrated sulfuric acid (H_2_SO_4_, 98%) and glycidoxypropyltrimethoxysilane (KH560) were supplied by Chengdu Kelong Chemical Reagent Factory, Chengdu, China. All the reagents were used without further purification. 

### 2.2. Surface Modification of HCNTs

**Figure 1:**
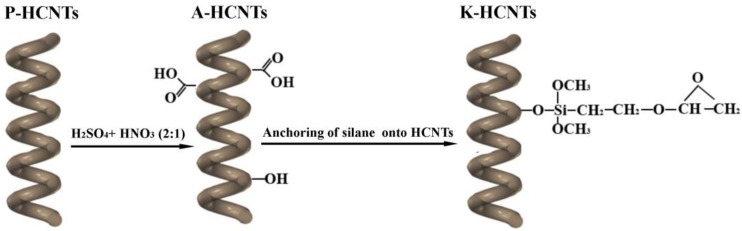
Schematic diagram for the acid oxidation and the silanization treatment of helical carbon nanotubes (HCNTs). Pristine HCNTs (P-HCNTs), acid treated HCNTs (A-HCNTs), and glycidoxypropyltrimethoxysilane (KH560) modified HCNTs (K-HCNTs).

In order to remove the amorphous carbon and organic impurities, the HCNTs were annealed at 700 °C for 2 h in Ar atmosphere. Figure 1 illustrates the procedures for the acid and the silanization treatments of HCNTs. Figure 1 shows that before the silanization treatment, the acid oxidation was first conducted to induce carbonyl groups on the surface of HCNTs. To achieve this, 0.3 g pristine HCNTs (P-HCNTs) were dispersed into a 2:1 (*v*/*v*) mixture of H_2_SO_4_/HNO_3_ using an ultrasonic bath at 40 °C for 1 h. Then, the acid treated HCNTs were collected by filtration and washed with deionized water until the pH of the filtrate reached neutral. The resultant HCNTs are defined as A-HCNTs. 

For the silanization of the A-HCNTs, the silane coupling agent (KH560) solution was prepared by mixing 7.5 mL KH560, 5 mL deionized water and 95 mL anhydrous ethanol and stirred continuously for 1 h to fully hydrolyse at pH = 5. Secondly, 0.2 g of A-HCNTs were treated with the KH560 solution for 3 h. Finally, the KH560-treated HCNTs were washed and dried at 60 °C for 24 h. The resultant materials are defined as K-HCNTs. It should be noted that the surface treatments of MWCNTs were exactly the same as those of HCNTs. The K-MWCNTs represent the silane treated MWCNTs.

### 2.3. Preparation of HCNT/Epoxy Composites

The HCNTs or MWCNTs were homogeneously dispersed into tetrahydrofuran (THF) by an ultrasonicator at high amplitude for 30 min. The dispersion was subsequently mixed with a certain amount of epoxy by using high shear mixing for 3 min at room temperature. The solvent (THF) was completely evaporated in an oven at 120 °C for 2 h. After that, the air bubbles and the residual THF were further removed at 120 °C for 2 h under vacuum. Then, the curing agent (DDS) was added into the epoxy/CNTs suspension and mixed on a magnetic stirrer at 150 °C for 30 min. Finally, the mixture was poured into Teflon molds and cured at 120 °C for 2 h, 160 °C 2 h and 180 °C for 2 h.

### 2.4. Characterizations

The surface chemical components of untreated and surface-treated HCNTs were analyzed by a Fourier transform infrared spectrometer (FTIR) (Nicolet 5700, Waltham, MA, USA) with a scanning range of 4000–400 cm^−1^.

X-ray photoelectron spectroscopy (XPS) was also used to study the chemical structure of HCNTs surface further. All XPS spectra were obtained using an Escalab 250Xi spectrometer (Thermo Fisher Scientific, Waltham, MA, USA) with a standard AlK X-ray source (200 W) and the pass analyzer pass energy of 30 eV.

In order to investigate the structural integrity of HCNTs before and after the surface modification, Raman spectra were recorded by a high-solution Raman spectrometer (inVia, Renshaw, London, UK) with laser excitation of 532 nm.

The morphology of HCNTs and the fracture surface of HCNT/epoxy composites were observed by using field-emission scanning electron microscopy (FE-SEM) (FE-SEM, JEOL, JSM-7001F) (Peabody, MA, USA) at accelerating voltage of 20 kV. The dispersion of HCNTs or CNTs in uncured epoxy was observed by a DM 2700 P optical microscope (LEICA, Wetzlar, Germany).

Thermogravimetry (TGA) measurements were performed on a thermo analysis system (STA449F3, NETZSCH, Selb, Germany). The temperature was increased from room temperature to 800 °C at 10 °C/min in the N_2_ atmosphere.

Tensile and flexural tests were conducted on a universal testing machine (CMT4304, SUST, Sansitaijie, Guangdong, China) at a crosshead speed of 2 mm/min, following the GB1040-92 and GB1449-2005, respectively. All the specimens were tested at room temperature. Six separate measurements were performed to provide the average value and the deviation.

## 3. Results and Discussion

### 3.1. Surface Modification of HCNTs

**Figure 2:**
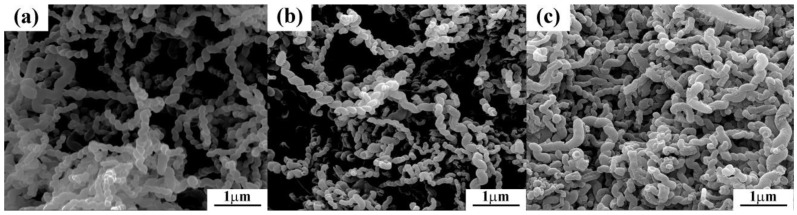
Scanning electron microscopy (SEM) images of P-HCNTs (**a**), A-HCNTs (**b**) and K-HCNTs (**c**).

The morphology of P-HCNTs, A-HCNTs and K-HCNTs was observed by using scanning electron microscopy (SEM) (Figure 2). As shown in Figure 2a, P-HCNTs with regular and homogeneous helical structure can be obtained by CVD. The surface of P-HCNTs is initially clear and smooth (Figure 2a). After the acid treatment, the morphology of A-HCNTs is not significantly changed in Figure 2b. However, the morphology of K-HCNTs becomes quite different (Figure 2c). The homogenous surface of K-HCNTs enveloped by a continuous and smooth KH560 layer is observed in Figure 2c. Compared to the P-HCNTs, the diameter of K-HCNTs is increased by 30% due to the presence of the KH560 thick layer. Thus, the silanization process endows the HCNTs with unique “core-shell” structure.

**Figure 3:**
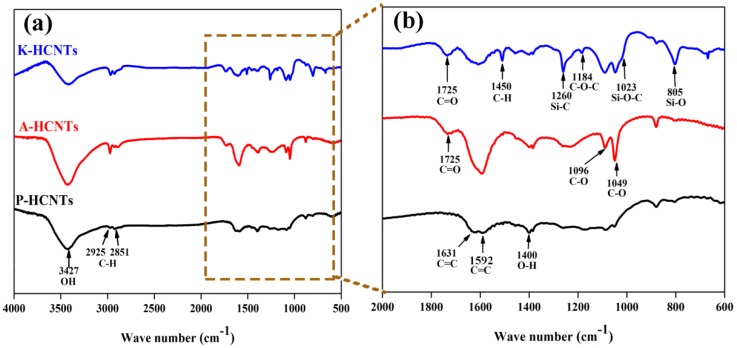
FTIR spectra (**a**) and magnified region of FTIR spectra from 2000 to 600 cm^−1^ (**b**) for P-HCNTs, A-HCNTs and K-HCNTs.

In order to detect the chemical groups of HCNTs induced by surface treatments, the Fourier-transfrom infrared spectra (FTIR) of P-HCNTs, A-HCNTs and K-HCNTs are shown in Figure 3. For the P-HCNTs, the peaks at 1400 and 3427 cm^−1^ represent the bending and stretching vibrations of hydroxyl groups (–OH), respectively. The presence of –OH is probably induced by the ambient atmospheric moisture or the oxidation during the purification of HCNTs [24,25]. The peaks of C–H and C=C originating from the carbon backbone of HCNTs are observed at 2925/2851 cm^−1^ (Figure 3a) and 1631/1592 cm^−1^ (Figure 3b), respectively. After the acid oxidation, the new peaks appear at 1725 cm^−1^, corresponding to the C=O stretching vibrations of the carboxylic groups (–COOH). It suggests that the surface of P-HCNTs is activated by the acid treatment. The peaks of C–O appear at 1049 and 1096 cm^−1^, resulting from the different alcohol structures on the HCNT surface. For the K-HCNTs, the new peaks of Si–O, Si–O–C and Si–C appear at 805, 1023 and 1260 cm^−1^, respectively. The peak of C–O–C and C–H from the KH560 molecules are also observed at 1184 and 1450 cm^−1^. Therefore, the presence of the KH560 molecules on the HCNT surface can be confirmed.

**Figure 4:**
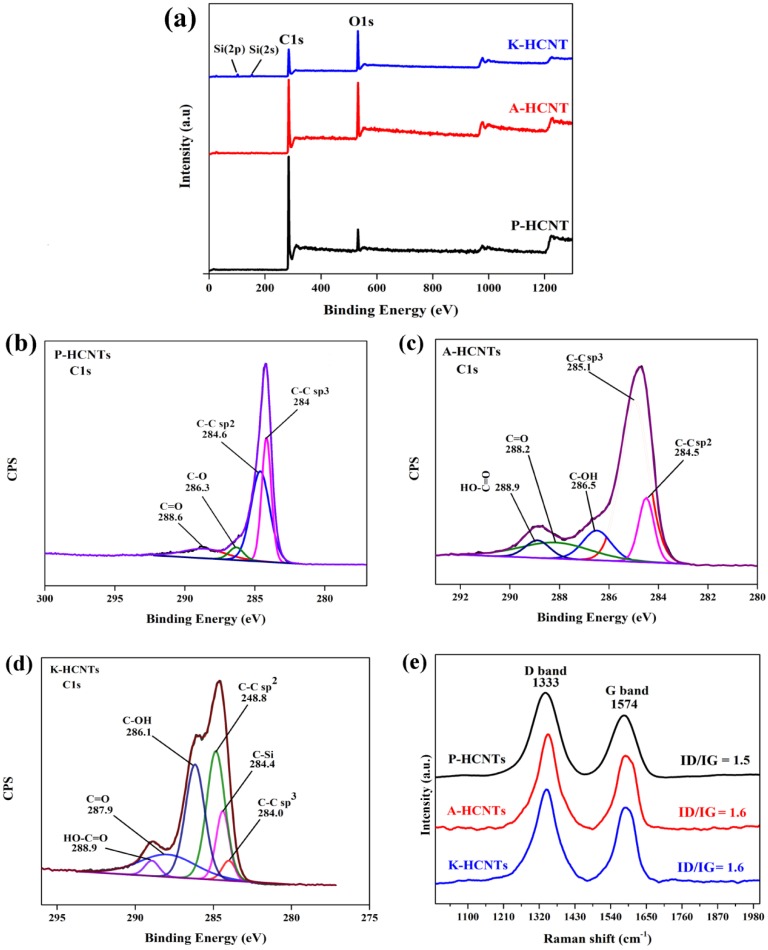
Effect of surface treatments on the surface chemical properties and the defect degree of HCNTs. X-ray photoelectron spectroscopy (XPS) survey spectra (**a**) of P-HCNTs, A-HCNTs and K-HCNTs. High-resolution XPS C1s spectra of P-HCNTs (**b**), A-HCNTs (**c**) and K-HCNTs (**d**). Raman spectra (**e**) of P-HCNTs, A-HCNTs and K-HCNTs.

To determine the surface elemental compositions of HCNTs after different surface treatments, the XPS survey spectra of P-HCNTs, A-HCNTs and K-HCNTs are shown in Figure 4a. As shown in Figure 4a, C1s and O1s peaks can be observed in every spectrum. Compared to the P-HCNTs, the density of O1s peaks of A-HCNTs and K-HCNTs is much higher, suggesting that various oxygen-containing functional groups are induced by the acid oxidation and the silanization treatments. In addition, there are two small Si 2s and Si 2p peaks observed in the spectrum of K-HCNTs, suggesting the presence of KH560 molecules grafted to the HCNTs. To further analyze the chemical compositions of untreated and surface-treated HCNTs, a more detailed investigation of the C1s peak is conducted and shown in Figure 4b–d. For the P-HCNTs (Figure 4b), the C1s peak can deconvoluted into four fitting curves originated from sp^3^ carbon atoms, sp^2^ carbon atoms, C–O and C=O, respectively. After the acid treatment, the peak of –COOH appears at 288.9 eV, meaning that the carboxyl groups are grafted on the surface of HCNTs (Figure 4c). In the spectrum of K-HCNTs (Figure 3e), a new peak of Si–C from KH560 molecules is observed at 284.4 eV after silanization, which is in good agreement with the FTIR results in Figure 3.

The defect degree of HCNTs before and after surface treatments was evaluated by Raman spectra (Figure 4e). It can be clearly seen from Figure 4e that two distinct peaks, namely G band and D band, appear at 1582 cm^−1^ and at 1341 cm^−1^ in this spectrum, respectively. The intensity ratio of the D band and the G band (*I_D_*/*I_G_*) can be used to represent the degree of CNT defects [26,27]. Figure 4e shows that the *I*_D_/*I*_G_ ratio is maintained after the surface treatments, which means that the graphitic integrity of HCNTs is not severely damaged by the acid oxidation or the silanization process. This is in full agreement with the SEM images (Figure 2) in which the morphology of HCNTs remains unchanged after different surface treatments.

### 3.2. Mechanical Properties of HCNTs/Epoxy Composites

**Figure 5:**
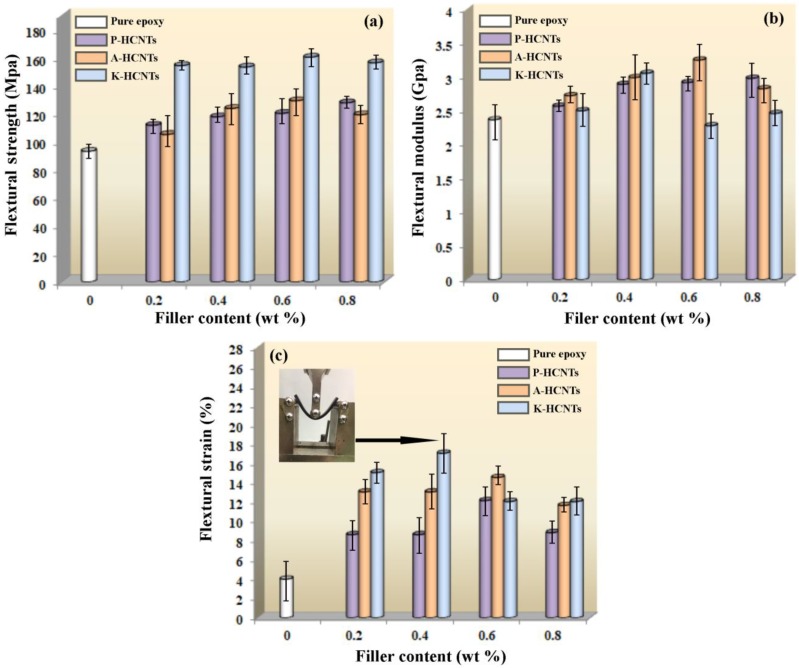
Flexural strength (**a**) and flexural modulus (**b**) and flexural strain (**c**) of pure epoxy, untreated HCNT/epoxy and surface-treated HCNT/epoxy composites as a function of HCNT content; the inset is an optical image exhibiting the high flexural strain of K-HCNT/epoxy composites during the bending test.

Figure 5 shows the flexural strength, the flexural modulus and the flexural strain of pure epoxy and HCNT/epoxy composites. It is found in Figure 5a that the addition of P-HCNTs and A-HCNTs slightly increases the flexural strength of HCNTs/epoxy composites, compared to pure epoxy. When the content of P-HCNTs is 0.8 wt %, the flexural strength reaches a maximum 128 MPa and increases by 37% compared with pure epoxy (93 MPa). For the A-HCNTs, the maximum improvement is about 39% with the addition of 0.6 wt % A-HCNTs. Compared to the P-HCNTs and the A-HCNTs, the contribution of K-HCNTs to the flexural strength of epoxy is much more notable. It is clear in Figure 5a that the flexural strength significantly increases with increasing K-HCNT content, up to the content of 0.6 wt %. In addition, the peak reaches at the HCNT content of 0.6 wt %, which is 72% higher than that of pure epoxy. However, the flexural modulus is slightly increased by adding untreated or surface-treated HCNTs (Figure 5b). Figure 5c illustrates that the flexural strain of pure epoxy, untreated HCNTs/epoxy and surface-treated HCNTs/epoxy composites versus the HCNT content. It is found that the flexural strain is significantly improved by the addition of HCNTs. The flexural deformation of HCNTs/epoxy composites increased from 4% for the pure epoxy to 12% and 15% for 0.6 wt % P-HCNTs and A-HCNTs, respectively. The maximum enhancement in the flexural strain is 200% and 263% for the P-HCNTs and A-HCNTs. After adding the K-HCNTs, the deformation of epoxy composites is remarkably improved and reaches a peak value at the HCNT content of 0.4 wt %. The flexural strain of K-HCNTs/epoxy composites can achieve 325% higher than that of pure epoxy. The inset of optical image in Figure 4c displays the high deformation of K-HCNTs/epoxy composites with 0.6 wt % K-HCNTs during bending. However, further increasing the HCNT content leads to the reduction of flexural properties, which is probably due to the formation of agglomerates [1].

**Figure 6:**
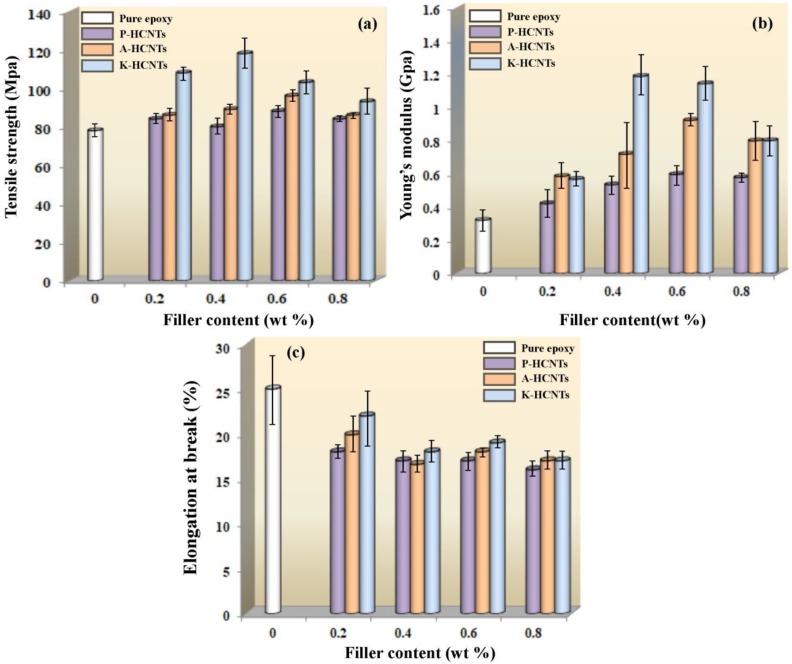
Tensile strength (**a**), Young’s modulus (**b**) and elongation at break (**c**) of pure epoxy, untreated HCNT/epoxy and surface-treated HCNT/epoxy composites.

Figure 6 displays the tensile properties of pure epoxy, untreated HCNT/epoxy and surface-treated HCNT/epoxy composites. Figure 6a shows that the addition of P-HCNTs and A-HCNTs slightly increases the tensile strength of epoxy. At the HCNT content of 0.6 wt %, the tensile strength of HCNT/epoxy composites increases by 12.8% for the P-HCNTs and 22.4% for the A-HCNTs compared to pure epoxy. For the K-HCNTs, the highest value is reached at a lower HCNT content, and the strengthening contribution is more remarkable. At the K-HCNT content of 0.4 wt %, the improvement in the tensile strength is around 513% higher than pure epoxy. Moreover, the addition of HCNTs significantly increases Young’s modulus of epoxy composites, especially for the K-HCNTs. As shown in Figure 6b, the optimal improvement in Young’s modulus is 86.3% for the 0.6 wt % P-HCNTs, 188% for the 0.6 wt % A-HCNTs and 270.9% for the 0.4 wt % K-HCNTs compared to pure epoxy. However, loading the HCNTs to epoxy leads to a decrease in the elongation at break of epoxy composites compared with pure epoxy (Figure 6c) and the elongation at break decreases as the HCNT concentration increases. This may result from the formation of HCNT aggregations that act as the mechanical defects for the resultant composites at high contents [28].

### 3.3. Strengthening and Toughening Mechanisms of HCNTs in Epoxy

**Figure 7:**
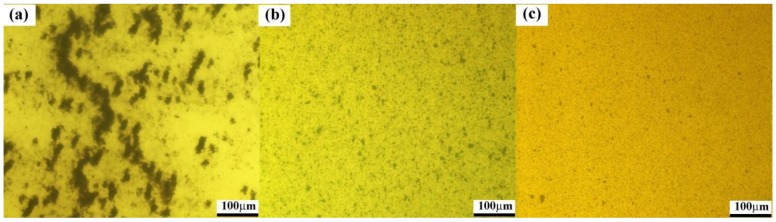
Optical microscopy images for the dispersion of P-HCNTs (**a**), A-HCNTs (**b**) and K-HCNTs (**c**) in uncured epoxy.

In order to explain the contribution of HCNTs to the flexural and tensile properties of HCNT/epoxy composites, the dispersion state of HCNTs in epoxy and the interfacial interaction between the HCNTs and epoxy were investigated by optical microscope and SEM, respectively. Figure 7 shows the optical microscopy images of P-HCNT, A-HCNT and K-HCNT/epoxy composites before curing. Before the surface treatments, the HCNTs tend to highly aggregate in epoxy and large areas of HCNT rich regions are observed (Figure 7a). After the acid treatment, the dispersion of A-HCNTs is significantly improved due to the presence of oxygen-containing functional groups on the HCNT surface. However, the HCNT clusters at micron scale still exist in epoxy as shown in Figure 7b. For the K-HCNTs (Figure 7c), the dispersion is further improved compared with the A-HCNTs due to the enhancement of the compatibility between the HCNTs and epoxy resin. In addition, the presence of the KH560 layer on the HCNTs induces a steric repulsion force to overcome the van der Waals forces between the HCNTs, thereby hindering the aggregations of HCNTs [20].

**Figure 8:**
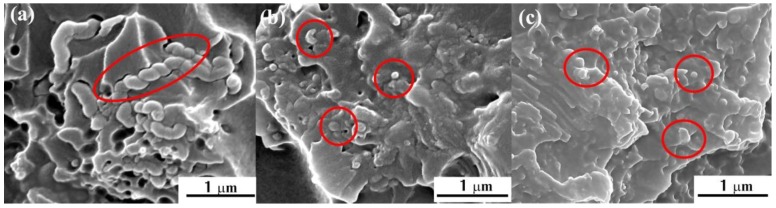
SEM images of the fracture surface of HCNT/epoxy composites with 0.6 wt % P-HCNTs (**a**), A-HCNTs (**b**), and K-HCNTs (**c**) after the three-point bending test. Red solid circles indicate the locations of HCNTs.

The interfacial interaction between the HCNTs and epoxy is investigated by SEM. Figure 8 presents the fracture surface of untreated HCNT/epoxy and surface-treated HCNT/epoxy composites. It is clearly seen in Figure 8a that there are many tiny holes left by the pulled out HCNTs and some voids at the HCNT/epoxy interface. In addition, the surface of pulled out HCNTs is clear and smooth, which suggests the poor interfacial interaction between the P-HCNTs and epoxy. After the acid treatment, the interfacial interaction between the A-HCNTs and epoxy is improved compared with the P-HCNTs as shown in Figure 8b. The A-HCNTs are slightly pulled out from the epoxy and have intimate contact with the epoxy matrix, resulting from the stronger interfacial interaction between them caused by the removal of impurities from the HCNTs and the introduction of functional groups after the acid treatment [19]. It is shown in Figure 8c that the interface between the K-HCNTs and epoxy is not discernable. In addition, the K-HCNTs are broken up instead of pulled out from the epoxy resin when the composite is subjected to the mechanical loadings, suggesting the highest interfacial strength between the K-HCNTs and epoxy. This is because that the KH560 layer plays a “bridging” role between the HCNTs and epoxy, thereby improving the interfacial interaction and promoting the interfacial stress transfer [1].

**Figure 9:**
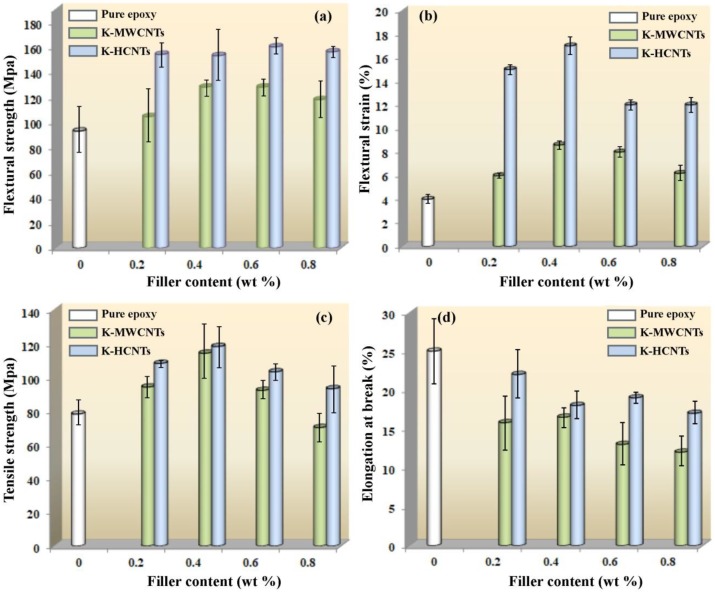
Comparison of the flexural and tensile properties of glycidoxypropyltrimethoxysilane (KH560) modified multi-walled carbon nanotube (K-MWCNT)/epoxy and K-HCNT/epoxy composites. Flexural strength (**a**), flexural strain (**b**), tensile strength (**c**), and elongation at break (**d**) of pure epoxy, K-MWCNT/epoxy and K-HCNT/epoxy composites as a function of the filler content.

To gain a deeper insight into the strengthening and toughening mechanisms of HCNTs, the effect of straight MWCNTs on the mechanical properties of epoxy composites was comparatively investigated. Figure 9 illustrates that the tensile and flexural properties of pure epoxy, K-MWCNT/epoxy and K-HCNT/epoxy composites. It is clearly shown in Figure 9a,b that the flexural strength and the flexural strain of K-HCNT/epoxy composites are higher than those of K-MWCNT/epoxy composites at all filler contents, which means that the contribution of K-HCNTs to the flexural properties is more significant than that of K-MWCNTs. The flexural strength and strain of K-HCNT/epoxy composites are 47.4% and 150.0% higher than those of K-MWCNT/epoxy composites at the filler content of 0.2 wt %. For the tensile properties, the tensile strength and the elongation at break of K-HCNT/epoxy composites are also higher than their K-MWCNT/epoxy counterparts at various filler contents (Figure 9c,d). It can be concluded that the HCNTs endow the epoxy composites with superior mechanical properties.

**Figure 10:**
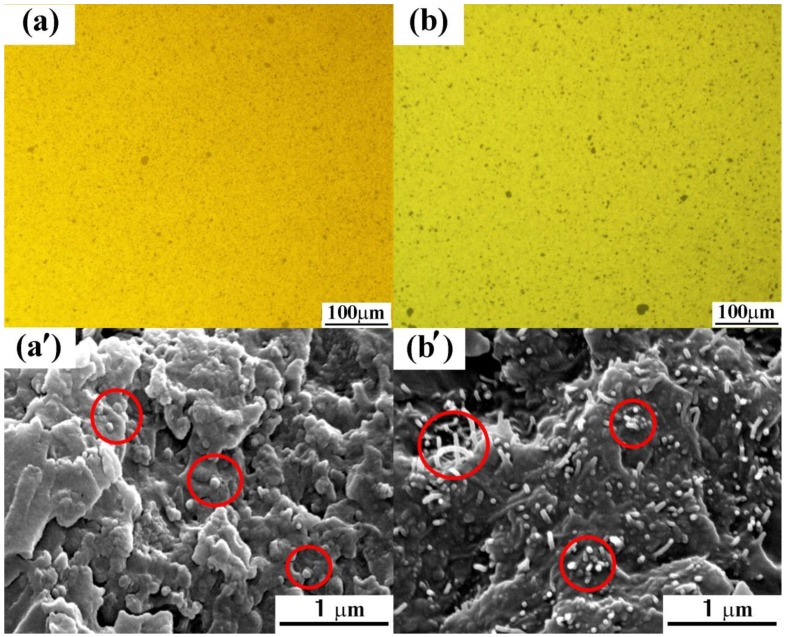
Microscope images of K-HCNT/epoxy (**a**) and K-MWCNT/epoxy composites (**b**) before curing. SEM images of the fracture surface of K-HCNT/epoxy (**a’**) and K-MWCNT/epoxy composites (**b’**) after the three-point bending test. The filler content of all the epoxy composites is 0.6 wt %. Red solid circles indicate the locations of HCNTs or MWCNTs.

To further explain the different effects of HCNTs and MWCNTs, the comparison of the dispersion of K-HCNTs and K-MWCNTs in epoxy and the interfacial interaction between them are presented in Figure 10. Large agglomerates of K-HCNTs and K-MWCNTs are not visible in Figure 10a,b. In addition, the dispersion states of K-HCNTs and K-MWCNTs in Figure 10a and b are comparative, which is supported by SEM images (Figure 10a’,b’). As mentioned above, the interfacial interaction between the K-HCNTs and epoxy is very strong (Figure 10a’), since the K-HCNTs are broken instead of being pulled out during the fracture of composites. However, it is clearly shown in Figure 10b’ that most of the K-MWCNTs are pulled out when the stress is subjected, suggesting the interfacial interaction between the straight CNTs and epoxy is weaker than that of HCNTs. It is generally believed that the HCNTs interlock tightly with epoxy due to their coiled morphology, which enhances the interfacial interaction between the HCNTs and epoxy and the mechanical properties of epoxy composites [21,22]. It should be noted that the geometry parameters of HCNTs, such as the coil parameters and the aspect ratio, are critical to the reinforcing and toughening effect on the mechanical properties of epoxy composites. Such research is ongoing in our group at present.

## 4. Conclusions

The effect of surface-treated HCNTs on the mechanical properties of epoxy composites was systematically investigated. The acid oxidation and the silanzation treatments were conducted to modify the HCNTs. The dispersion of HCNTs in epoxy and the interfacial interaction between the HCNTs and epoxy were significantly improved by these treatments. The flexural strength and the flexural strain of K-HCNT/epoxy composites are 72% and 325% higher than those of pure epoxy. In addition, the tensile strength and Young’s modulus of epoxy composites were simultaneously enhanced by 51% and 270.9% after combining with the K-HCNTs. This is because the presence of KH560 molecules promotes the dispersion of HCNTs in epoxy and enhances the interfacial interaction. Compared with the straight MWCNTs, the coiled HCNTs exhibit superior strengthening and toughening contributions to the epoxy composites, resulting from the mechanical interlocking between the coils of HCNTs and epoxy.
